# Spontaneous Pneumothorax Secondary to Bullous Lung Emphysema Positive for Cannabinoids upon Toxicological Examination

**DOI:** 10.3390/jcm12154956

**Published:** 2023-07-28

**Authors:** Mario Bisconti, Angela De Palma, Roberta Pacifici, Maria Concetta Rotolo, Simona Pichini, Debora Brascia, Xenia Trabucco, Manuela Pellegrini, Laura Carrozzi, Francesco Pistelli, Giuseppe Marulli

**Affiliations:** 1 Unit of Thoracic Surgery, Department of Precision and Regenerative Medicine and Ionian Area, University of Bari “Aldo Moro”, Piazza Giulio Cesare 11, 70124 Bari, Italy; drbiscontimario@gmail.com (M.B.); deborabrascia@gmail.com (D.B.); giuseppe.marulli@uniba.it (G.M.); 2 Unit of Pneumology, Hospital “Vito Fazzi”, 73100 Lecce, Italy; 3 National Centre on Addiction and Doping, Istituto Superiore di Sanità, 00161 Rome, Italy; roberta.pacifici@iss.it (R.P.); mariaconcetta.rotolo@iss.it (M.C.R.); simona.pichini@iss.it (S.P.); manuela.pellegrini@iss.it (M.P.); 4 Unit of Pathology, Department of Precision and Regenerative Medicine and Ionian Area, University of Bari “Aldo Moro”, 70124 Bari, Italy; xeniatrabucco@hotmail.it; 5 Unit of Pneumology, University of Pisa, 56126 Pisa, Italy; laura.carrozzi@unipi.it (L.C.); f.pistelli@ao-pisa.toscana.it (F.P.)

**Keywords:** cannabis smoking, cannabinoids, delta-9-tetrahydrocannabinol (THC), primary spontaneous pneumothorax (PSP), bullous lung emphysema

## Abstract

Cannabis can be related to respiratory diseases, but the relationship between smoking marijuana and the development of a pneumothorax has scarcely been investigated. We aimed to analyze, in patients with a history of cannabis smoking abuse submitted to lung apicectomy for a primary spontaneous pneumothorax (PSP), the correlation between the presence of cannabinoids in the resected lung and the detection of bullous emphysema within the same tissue. Patients undergoing lung apicectomy for a PSP were prospectively enrolled, and the correlation between the presence of cannabinoids in the resected lung tissue and histological finding of bullous emphysema was investigated with Fisher’s exact test. There were 21 male patients, with a median age of 27 years. The cannabinoids found by the toxicological examination in surgical specimens were mainly delta-9-tetrahydrocannabinol (THC), cannabinol (CBN), and cannabidiol (CBD). In 14/21 patients, cannabinoids were detected in the resected lung tissue, and bullous emphysema was present in 13/14 of these (93%), while bullous emphysema was found in only 1/7 (14%) of the remaining patients who were negative for cannabinoids in the lung tissue, and the difference was found to be statistically significant (*p* < 0.0009). Our study demonstrated the presence of bullous emphysema in most cannabinoid-positive patients and its absence in most of those who were cannabinoid-negative, supporting the correlation between cannabinoids in the lung tissue and bullous emphysema with the development of a “secondary” spontaneous pneumothorax.

## 1. Introduction

Although respiratory diseases caused by drugs (Re.Di.D.) have a long history of occurrence, most cases were reported in the second half of the twentieth century [1]. Recently, in chronic tobacco and marijuana smokers, chest computed tomography (CT) evidence of emphysema bullae, centrilobular emphysema, and paraseptal emphysema has been reported [2]. Other studies have suggested an association between tobacco and cannabis smoking and the development of bullous lung emphysema with a primary spontaneous pneumothorax (PSP) [1,3,4,5,6,7].

Cannabinoids are detectable in the urine, hair, and blood. In clinical practice, a temporal correlation between substance consumption and the onset of a respiratory disease can suggest a causal effect when the substance is detected in the urine or blood, while the diagnosis is only presumptive if the substance is not identified in lung tissue.

Thus, in our previous studies, we developed a method to identify cannabinoids in the bronchoalveolar lavages and lung tissues of cannabis smokers, and in our last study we compared patients with and without a history of cannabis abuse treated with apicectomy for a PSP, supporting a correlation between this disease and cannabis abuse [8,9].

The next step of our research was to detect significant histological abnormalities in lung tissues positive for cannabinoids, such as the presence of bullous emphysema, in order to demonstrate the role of cannabis in the pathogenesis of pneumothorax.

Therefore, a prospective review of patients with a history of cannabis smoking undergoing the mini-invasive wedge resection of lung tissue for a PSP (indications: persistent pneumothorax after chest tube; evidence of bullous disease on CT; recurrent pneumothorax) was conducted. An evaluation of the presence of cannabis byproducts within the resected specimens was performed, and the correlation between the presence of cannabinoids and the development of bullous emphysema was investigated.

The objective of the study was to analyze the prevalence of bullous emphysema in resected lung tissue positive for cannabinoids according to a toxicological examination compared to resected lung tissue negative for cannabinoids.

Finding a correlation between the presence of cannabinoids in the resected lung and the detection of bullous emphysema within the same tissue would support the role of cannabinoids in the pathogenesis of lung bullous emphysema with the development of a “secondary” spontaneous pneumothorax caused by cannabis.

As marijuana is a plant that can be classified into two varieties, cannabis indica and cannabis sativa, originating from different geographical areas, the words marijuana and cannabis are used as synonyms.

## 2. Materials and Methods

### 2.1. Population of the Study

Patients aged over 18 years with a diagnosis of a PSP were prospectively enrolled. The inclusion criteria were a PSP requiring surgical treatment (persistent pneumothorax after chest tube insertion or with chest CT evidence of lung bullous idiopathic dystrophy or recurrent pneumothorax) and a current or previous history of drug abuse (cannabis smoking, associated or not with tobacco smoking).

Patients were asked about: their consumption of cocaine, heroin, ecstasy, and other substances; the number of tobacco and/or cannabis cigarettes smoked per day; and the time of their last consumption of these substances before the onset of pneumothorax symptoms (chest pain, cough, dyspnoea).

Surgical treatment, performed in the same thoracic unit for all patients, consisted in a wedge resection of the lung apex (“apicectomy”, including apical blebs and/or bullous dystrophy) and pleurodesis via video-assisted thoracic surgery (VATS); in some cases, evident blebs in other lobes were also resected.

The study was conducted in accordance with the Declaration of Helsinki (as revised in 2013) and was approved by the ethics board of the Hospital “Vito Fazzi”, Lecce, Italy (6 June 2019/no.33).

In addition to the standard surgery consent, written informed consent was obtained from each patient for the toxicological examination of their pleuro-pulmonary tissue.

Two specimens were taken from each patient: one was sent to the Unit of Pathology for a standard pathological examination, and the second was frozen (−18 °C) and sent to the National Centre on Addiction and Doping for toxicological examination and the identification of cannabinoids in lung tissue.

### 2.2. Methods for Identification of Cannabinoids in Lung Tissue

Detailed specifications of the methods used for the identification of cannabinoids in lung tissue [9] are here reported.

For the extraction of cannabinoids from the lung [9], 300 μL of 0.1 N NaOH and 3 mL of hexane/ethyl acetate (9:1) were added to one gram of the pulmonary tissue specimen. The solvent was then evaporated to dryness.

Trimethylsilyl derivatives were prepared and then analyzed by gas chromatography/mass spectrometry. Analyses were carried out on a 6890 Series Plus gas chromatograph equipped with an Agilent 7683 autosampler and coupled to a 5973 N mass selective detector (Agilent Technologies, Palo Alto, CA, USA).

Data acquisition and analysis were performed using standard software supplied by the manufacturer (Agilent Chemstation, Palo Alto, CA, USA).

Analyte separation was achieved in a fused silica capillary column (HP-5MS, 30 m, 0.25 mm i.d, film thickness 0.25 mm) (Agilent Technologies).

The oven temperature was programed at 140 °C for 2 min, increased to 290 °C at 20 °C/min, and held for 10 min.

The split injection mode (15:1) was applied.

For quantitative analysis, the following ions were monitored: *m*/*z* 303, 371, and 386 for the THC-trimethylsilyl derivative; *m*/*z* 310, 367, and 382 for the CBN-trimethylsilyl derivative; *m*/*z* 301, 337, and 390 for the CBD-trimethylsilyl derivative; *m*/*z* 371, 459, and 474 for the 11-OH-THC-trimethylsilyl derivative; *m*/*z* 371, 473, and 488 for the THC-COOH-trimethylsilyl derivative; *m*/*z* 306, 374, and 389 for the delta-9-THC-trimethylsilyl-d3 derivative; and *m*/*z* 374, 476, and 491 for the THC-COOH-trimethylsilyl-d3 derivative.

### 2.3. Statistical Analysis

Continuous variables are reported as mean with standard deviation (SD) and median with range. Nominal variables are reported as counts and percentages. For dichotomous variables (association between the presence of cannabinoids in the resected lung tissue and the presence of bullous emphysema or other pathologic characteristics), the non-parametric Fisher’s exact test was used (RStudio, R v3.6.2, Dark and Stormy Night); *p*-values < 0.05 were considered significant.

## 3. Results

Twenty-one male patients were enrolled and submitted to surgical treatment in the form of a lung apicectomy for a PSP. The mean age was 30 ± 9.24 years, and the median age was 27 years (range: 18–51).

Concerning the indications for surgery, in 11 patients there was evidence of bullous disease in the chest CT scan; in 5 a patients persistent pneumothorax was present after a chest tube (in two cases, bullous disease was also found in the chest CT scan); and in 5 patients a recurrent pneumothorax (3 homolateral and 2 contralateral) occurred.

Among the 21 patients, in additional to cannabis use, 2 patients also reported both cocaine and heroin use, another 2 patients cocaine use, and 1 other patient heroin use; 19 patients out of 21 were also tobacco smokers (Table 1).

Of the four patients also reporting the use of cocaine, it was consumed via inhalation by three and via inhalation and per os by one; the three patients also reporting the use of heroin took it intravenously.

In 14/21 (67%) patients, cannabinoids were detected in the resected lung tissue, while 7/21 (33%) were found to be negative for cannabinoids in the lung tissue.

Table 2 reports the declared numbers of tobacco cigarettes and cannabis cigarettes smoked and the time between the last cannabis consumption and the onset of the pneumothorax in relation to the results of the toxicological examination (the presence or absence of cannabinoids in the resected lung tissue). The presence or absence of bullous emphysema in the resected lung tissue, as well as the presence of other pathologic characteristics, are also reported in relation to the results of the toxicological examination.

Among the 14 patients with lung tissue positive for cannabinoids, we observed that 11 (79%) declared that their last cannabis consumption was from 30 min to 1 month before PSP onset, and 3 (21%) declared that it was from 2 to 8 months before PSP onset.

On the other hand, among the seven patients with lung tissue negative for cannabinoids, five (71%) declared that their last cannabis consumption was from 1 year to 3 years before PSP onset.

Although some patients declared that they had also used cocaine and heroin, these substances were not detected in the resected lung tissue.

**Figure 1:**
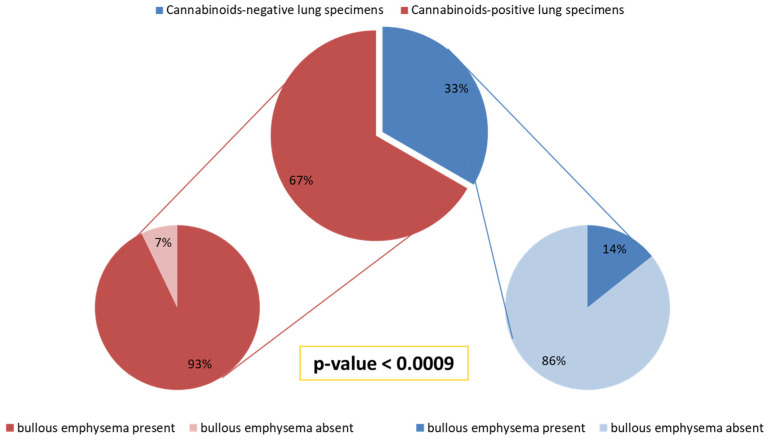
Correlation between presence of cannabinoids in lung specimens and histological findings of bullous emphysema: bullous emphysema was present in 13/14 (93%) subjects with cannabinoid-positive lung specimens, while it was found in only 1/7 (14%) of the remaining patients with cannabinoid-negative lung specimens, and the difference was statistically significant.

Bullous emphysema was present in 13/14 (93%) subjects with lung tissue positive for cannabinoids (THC, CBN, CBD), while only 1/7 (14%) of the remaining subjects with resected lung tissue negative for cannabinoids (THC, CBN, CBD) had bullous emphysema (*p* < 0.0009) (Figure 1).

Moreover, we found that 9/13 (69%) of subjects positive for cannabinoids in their lung tissue and with bullous emphysema smoked one cannabis cigarette per day, and all 9 patients were tobacco smokers, too.

On the other hand, 3/6 (50%) subjects negative for cannabinoids in their lung tissue and without bullous emphysema smoked one cannabis cigarette per month, and all 3 patients were tobacco smokers, too.

Furthermore, upon pathological examination, pleural-interstitial fibrosis was detected in 12/14 (86%) patients with lung tissue positive for cannabinoids, non-specific chronic inflammation in 9/14 (64%), and a desquamative interstitial pneumonia (DIP)-like reaction in 7/14 (50%). Among the patients who presented lung tissue negative for cannabinoids but declared that they were cannabis smokers, pleural-interstitial fibrosis was present in 5/7 (71%), non-specific chronic inflammation in 6/7 (86%), and a DIP-like reaction in 2/7 (29%).

No significant association was found between the presence of cannabinoids in the resected lung tissue and the presence of each of these pathological findings compared with the negative lungs.

Only five patients underwent a blood test to detect the values of the alpha-1-antitrypsin enzyme, which were found to be normal.

## 4. Discussion

Different studies have suggested a correlation between marijuana smoking and the development of bullous emphysema. Johnson et al. described four young males with significant marijuana exposure but low tobacco exposure who were affected by large lung bullae [10]. Hii et al. reported that in a series of 10 regular chronic marijuana smokers (more than one year, continuously), 4 had a pneumothorax, and high-resolution chest CT revealed asymmetrical, variably sized, emphysematous bullae in 9 of them [11]. Fiorelli et al. found lung emphysematous bullae in 8/13 (62%) habitual marijuana smokers [12]. Habitual marijuana smokers are defined as users of more than 100 marijuana cigarettes (joints) during their lifetime and at least one in the previous month [13]. Feldman et al. described a case with an apical lung bulla in a marijuana and tobacco smoker [14]. Rawlins et al. reported two marijuana users, male patients with bilateral giant lung bullae [15]. In a study by Douglass et al., of 525 diagnoses of a pneumothorax, 113 (21.5%) occurred as a result of drug abuse in 84 patients [16].

Moreover, other recent studies have underlined that: the combination of smoking cannabis and tobacco significantly increases the risk of having a PSP in young men compared to both those who have never smoked and daily tobacco smokers [17]; cannabis smokers develop more severe chronic respiratory symptoms and bullous lung disease than both tobacco smokers and non-smokers [18]; cannabis use is associated with PSP recurrence and the eventual need for operative intervention [19].

Furthermore, in our other study we identified, for the first time in the literature, the principal cannabinoids in the lungs of a group of young patients who underwent operations for a PSP and were former or current tobacco smokers with a history of cannabis consumption. Additionally, we compared these patients with a group of patients of the same mean age without a history of cannabis abuse who were surgically treated for a PSP, supporting a correlation between this disease and cannabis abuse [9].

Our finding of these drugs in lung tissue opened the way for the study of their pathogenic mechanisms.

A previous study by Beshay et al. [4] reported the largest case series of spontaneous pneumothorax in habitual marijuana smokers with upper-lobe severe bullous lung disease and therefore suggested considering cannabis abuse as a cause of emphysema in young patients.

However, the mechanism for bullae development in cannabis smokers is not well-known, and various hypotheses have been put forward: the direct action of toxic components in marijuana, prolonged breath holding producing high inspiratory pressure, and the activation of inflammatory cells triggering the loss of lung elastic recoil [9,12]. It should also be noted that in marijuana smokers, barotraumas with a consequent pneumothorax, pneumomediastinum, and subcutaneous emphysema may occur with deep inhalation involving breath holding or Valsalva maneuvers [9].

Nevertheless, while suggesting the responsibility of cannabis for the development of bullous lung disease in young subjects and the consequent risk of a pneumothorax, none of the aforementioned studies examined the presence of cannabinoids in lung tissues and related pathologic findings of bullous emphysema. Thus, our research was innovative, investigating this correlation for the first time.

In our study, we described 21 male patients who underwent operations for a PSP and had a history of cannabis smoking (in 19/21 cases, this was associated with tobacco smoking).

Cannabinoids were detected in the lung tissue of about 67% of the patients, and bullous emphysema was present in 93%. Of the patients, 69% smoked one cannabis cigarette per day, and all were tobacco smokers too, showing that in young people even a single cannabis cigarette smoked per day seems to contribute to the development of bullous emphysema, especially if accompanied by tobacco smoking.

On the other hand, in most patients (86%) with lung tissue negative for cannabinoids, no bullous emphysema was found, and 50% of these patients smoked one cannabis cigarette per month and were tobacco smokers too, suggesting that smoking fewer cannabis cigarettes is related to a lower risk of developing bullous emphysema, even if accompanied by tobacco smoking.

The evaluation of the association between the presence of cannabinoids in resected lung tissue and the presence of bullous emphysema produced statistically significant results (*p* < 0.0009), thus supporting (although the prevalence of bullous emphysema in cannabis-only smokers is unknown [20]) the responsibility of cannabis for the development of bullous emphysema, as hypothesized in other studies [2,10,11,12,13,14,15,16]. However, the presence of cannabinoids in lung tissue was not determined in these studies.

The retention time of cannabinoids in lung tissue was calculated by considering the declared last instance of cannabis consumption. In 12/14 (86%) patients with lung tissue positive for cannabinoids, the last consumption of cannabis was <3 months before PSP onset, and in 2/14 (14%) it ranged from 3 to 12 months before PSP onset, while in 5/7 (71%) patients with lung tissue negative for cannabinoids, it varied from 1 to 3 years before PSP onset. This observation suggested that the maximum retention time of cannabinoids in lung tissue is about 1 year, while in the urine of chronic smokers, it can remain for about 77 days [21]. Regarding the other 2/7 (29%) patients negative for cannabinoids who reported that they had last consumed cannabis < 3 months prior to PSP onset, these were patients with psychiatric disorders, and so they may have not provided totally reliable historical data.

Although some patients had also used cocaine and heroin, these substances were not detected in the resected lung tissue, likely due to their more rapid elimination compared to cannabinoids.

Most of our patients were also tobacco smokers, but since emphysema usually develops at around 50–60 years in tobacco smokers, while bullous emphysema appeared at a younger age in the studied patients, we believe that cannabis has a decisive impact in its development, while tobacco smoking has a minor role at a younger age. Moreover, the fact that we identified patients who were negative for cannabinoids in the lung tissue and did not present bullous emphysema but smoked 5 to 50 tobacco cigarettes per day supported the responsibility of cannabis for the development of bullous emphysema in these young patients. The study by Bense et al. supports our thesis, reporting that, in 27 chronic tobacco smokers with a previous spontaneous pneumothorax, chest CT revealed the absence of bullous emphysema in all, the presence of areas of focal emphysema in 22 (81%), and the absence of emphysema in 10 non-smoking control subjects [22]. Moreover, in our patients, other diseases characterized by the presence of bullous emphysema, such as Marfan syndrome, Ehlers–Danlos syndrome, HIV infection, and the intravenous use of illicit drugs, were absent. Furthermore, as all patients in our study were young and healthy, only five of them underwent a blood test to detect the values of the alpha-1-antitrypsin enzyme, which turned out to be normal.

In reference, then, to the rupture of bullae, we believe that this is favored not only by the deep inhalation used by cannabis smokers [23,24] who, in order to increase the absorption surface of the airways, practice the Valsalva maneuver [16,24,25,26], but also by the inflammatory process, evidence of which we found during the histological examination of the operative specimens. In fact, pleural-interstitial fibrosis, non-specific chronic inflammation, and DIP-like reactions were detected in the majority of patients with lung tissue positive for cannabinoids.

Hartman [27] and Carrington [28] suggested the responsibility of marijuana for the genesis of desquamative interstitial pneumonia (DIP); in some of our patients (7/14 cannabinoid-positive and 2/7 cannabinoid-negative patients), a DIP-like reaction was detected in the lung tissue. Thus, in our study, the role of cannabis smoking in causing DIP-type lung lesions was supported by not only the age of our patients (ranging from 18 to 51 years, while for DIP induced by other causes, the age usually ranges from 40 to 60 years [29]) but also the finding of a large number of macrophages within the alveoli. In fact, DIP-like reactions are characterized by the accumulation of many macrophages, presenting a light brown pigment, in the alveolar spaces.

When lung tissue is damaged by the chronic irritative insult caused by tobacco and cannabis smoke, neutrophils and monocytes are activated, with the release of toxic factors, such as proteases, elastases, and collagenases. This chronic irritative stimulus could be an etiopathogenetic factor favoring the formation of emphysematous bullae. This process, as emerged in our study, could occur earlier in patients with cannabinoid-positive lung tissue. The endoalveolar presence of macrophages (DIP-like reactions), histiocytes, or foreign-body giant cells, indicative of a chronic inflammatory process, supports the role of cannabinoids in favoring the chronic non-specific inflammatory process with the secondary lysis of the interalveolar septa, which in turn become the sites of the chronic inflammatory process with interstitial fibrosis and the consequent formation of bullous emphysema. Therefore, in our study, the factor that differentiated cannabinoid-positive subjects from cannabinoid-negative ones, with all subjects being drug users, was the time of the development of bullous emphysema, and both groups presented the same pathogenetic mechanism, as was also suggested by Cagle et al. [30].

With reference, then, to concomitant tobacco smoke, Gong et al. report a study comparing subjects between 25 and 45 years of age (non-smokers, tobacco smokers, marijuana smokers, and tobacco and marijuana smokers) and concluded that habitual, heavy marijuana smokers had a high prevalence of abnormal airway appearance and histologic findings, irrespective of concomitant tobacco smoking; they also found that smoking 3–4 joints per day produced in the airways the same histological changes as smoking 20 tobacco cigarettes per day [31].

According to some authors [16], symptoms of a pneumothorax generally appear within 24 h from the consumption of illicit drugs, as we also observed in 7/14 (50%) of the cannabinoid-positive patients and in only 1/7 (14%) of the cannabinoid-negative patients. Therefore, considering their positivity for cannabinoids in the resected lung tissue, the pneumothorax with bullous emphysema we observed in these young patients was not considered of unknown cause (primary) but secondary to cannabis.

This study had some limitations: some of our patients declared the use of other illicit drugs, and their possible role and contribution to the development of bullous emphysema was not investigated; positivity for cannabinoids in the lung tissue was not correlated to the eventual presence of cannabinoids in the urine and/or blood, as this was not investigated in these patients; most of our patients were active tobacco smokers, which could have been a confounding or contributing factor in the pathologic findings of bullous emphysema; we encountered difficulties in recruiting patients, who were reluctant to provide informed consent for the toxicological examination of their lung tissue.

## 5. Conclusions

In conclusion, our study not only supported the previous literature that, only on the basis of the chest CT detection of bullous emphysema in cannabis smokers, hypothesized its correlation with cannabis smoking, but also demonstrated a direct relationship between cannabis smoking and bullous emphysema, having found cannabinoids and bullous emphysema in the resected lung tissue of young patients who underwent operations for a pneumothorax.

Thus, by demonstrating a correlation between cannabinoids in resected lung tissue and bullous emphysema, this study ratified the responsibility of cannabis for the formation of bullous emphysema, defining, for the first time in the literature, a spontaneous pneumothorax secondary to cannabis-induced bullous emphysema. Furthermore, our results suggest that in clinical practice, in the case of surgery, the presence of this substance in the resected lung tissue should be determined in order to make a correct diagnosis of a pneumothorax secondary to cannabis. In this regard, the temporal correlation between the intake of cannabis and the onset of a pneumothorax is no longer sufficient for diagnosis, even when the substance is present in the urine or blood, if it is not found in the lungs, where cannabinoids can remain from the moment of intake for as long as a year.

## Figures and Tables

**Table 1:** Characteristics of patients who underwent PSP operations and the substances they used.

**Number of Patients**	**21**
Male:female	21:0
Median age in years (range)	27 (18–51)
**History of Abuse**	
Tobacco	19 (90%)
Cannabis	21 (100%)
Cocaine	4 (19%)
Heroin	3 (14%)

**Table 2:** Specification of patients’ declared number of tobacco cigarettes and cannabis cigarettes smoked compared to the results of the toxicological examination and the presence or absence of bullous emphysema in the resected lung tissue; time between last cannabis consumption and onset of PSP and presence of other pathological findings are also reported.

Patient n.	No. Tobacco Cigarettes/Day	No. Cannabis Cigarettes/Day	Time between Last Cannabis Consumption and Onset of PSP	THC, CBN, and CBD in Lung Tissue	Bullous Emphysema	Pleural- Interstitial Fibrosis	Non-Specific Chronic Inflammation	DIP-Like Reaction
1	10	1	1 day	+	+	+	+	-
2	20	1/month	1 month	+	+	+	-	+
3	20	1	15 days	+	+	+	-	+
4	15	1	30 min	+	+	+	+	+
5	10	1/month	3 years	-	-	+	-	-
6	20	1	30 min	+	+	+	+	-
7	20	1	2 months	+	+	+	+	+
8	5	1/month	2 months	-	-	+	+	-
9	20	1	3 days	+	+	+	-	-
10	15	1	4 months	+	+	+	+	-
11	3–5	1	30 min	+	+	+	-	+
12	7–9	2	3 h	+	-	+	+	-
13	15	1/month	96 h	-	-	-	+	-
14	10	1/month	1 month	+	+	-	+	+
15	none	1/month	96 h	+	+	+	+	+
16	10	1	1 year	-	-	+	+	+
17	4	1	1 year	-	+	-	+	-
18	50	1	2 years	-	-	+	+	+
19	5	1/month	8 months	+	+	+	-	-
20	3–4	1–2	1 day	+	+	-	+	-
21	none	1	1 year	-	-	+	+	-

No. = number; THC = delta-9-tetrahydrocannabinol; CBN = cannabinol; CBD = cannabidiol; + = present; - = absent.

## Data Availability

The datasets used and/or analyzed during the current study are available from the corresponding author upon reasonable request.
